# Limitations of Rapid Diagnostic Testing in Patients with Suspected Malaria: A Diagnostic Accuracy Evaluation from Swaziland, a Low-Endemicity Country Aiming for Malaria Elimination

**DOI:** 10.1093/cid/cix131

**Published:** 2017-03-27

**Authors:** Nikhil Ranadive, Simon Kunene, Sarah Darteh, Nyasatu Ntshalintshali, Nomcebo Nhlabathi, Nomcebo Dlamini, Stanley Chitundu, Manik Saini, Maxwell Murphy, Adam Soble, Alanna Schwartz, Bryan Greenhouse, Michelle S. Hsiang

**Affiliations:** 1 Malaria Elimination Initiative, Global Health Group,; 2 Global Health Sciences, and; 3 Department of Medicine, University of California, San Francisco;; 4 Department of Pediatrics, Benioff Children’s Hospital and University of California, San Francisco;; 5 Emory University School of Medicine, Atlanta, Georgia;; 6 Department of Pediatrics, University of Texas Southwestern Medical Center, Dallas;; 7 National Malaria Control Programme and; 8 Clinton Health Access Initiative, Mbabane, Swaziland

**Keywords:** malaria, Rapid Diagnostic Test, diagnostic accuracy, low transmission, subpatent infection.

## Abstract

**Background.:**

The performance of *Plasmodium falciparum*–specific histidine-rich protein 2–based rapid diagnostic tests (RDTs) to evaluate suspected malaria in low-endemicity settings has not been well characterized.

**Methods.:**

Using dried blood spot samples from patients with suspected malaria at 37 health facilities from 2012 to 2014 in the low-endemicity country of Swaziland, we investigated the diagnostic accuracy of histidine-rich protein 2–based RDTs using qualitative polymerase chain reaction (PCR) (nested PCR targeting the cytochrome *b* gene) and quantitative PCR as reference standards. To explore reasons for false-negative and/or false-positive results, we used *pfhrp2/3*-specific PCR and logistic regression analyses of potentially associated epidemiological factors.

**Results.:**

From 1353 patients, 93.0% of RDT-positive (n = 185) and 31.2% of RDT-negative samples (n = 340) were available and selected for testing. Compared with nested PCR, the sensitivity, specificity, positive predictive value (PPV), and negative predictive value (NPV) of RDTs were 51.7%, 94.1%, 67.3%, and 89.1%, respectively. After exclusion of samples with parasite densities <100/μL, which accounted for 75.7% of false-negative results and 33.3% of PCR-detectable infections, the sensitivity, specificity, PPV, and NPV were 78.8%, 93.7%, 62.3%, and 97.1%. Deletions of *pfhrp2* were not detected. False-positivity was more likely during the second year and was not associated with demographics, recent malaria, health facility testing characteristics, or potential DNA degradation.

**Conclusions.:**

In the low-transmission setting of Swaziland, we demonstrated low sensitivity of RDT for malaria diagnosis, owing to an unexpectedly high proportion of low-density infection among symptomatic subjects. The PPV was also low, requiring further investigation. A more accurate point-of-care diagnostic may be needed to support malaria elimination efforts.

## Summary

In a low-endemicity/malaria elimination setting, we demonstrate limited sensitivity of *Plasmodium falciparum*–specific rapid diagnostic testing for suspected malaria, owing to unexpected low-density infections. Positive predictive value was also low, requiring further investigation. More accurate diagnostics may be needed.

Between 2000 and 2015, the worldwide incidence rate of malaria dropped by 37% [1]. Most of this success has been attributed to improved vector control, therapeutics, and diagnostics [2]. Increased diagnostic testing since 2005 has followed a surge in procurement by national governments of rapid diagnostic tests (RDTs), which are immune chromatography–based assays that detect malaria antigens, such as *Plasmodium falciparum*– specific histidine-rich protein 2 (HRP-2) [1, 3–7]. RDTs have revolutionized malaria diagnosis by providing convenience and a rapid turn-around time of only 15–20 minutes. Demand for RDTs has grown in the last decade from 46 million tests sold in 2008 to 314 million sold in 2014 [8].

Accurate diagnosis of malaria is critical for appropriate patient management and population level surveillance. Early studies of *P. falciparum* HRP-2–based RDT performance were mainly from moderate- and high-transmission settings (parasite prevalence by polymerase chain reaction (PCR), ≥10% [9, 10]) and showed reliable sensitivity and specificity of 93.5%–96.2% and 93.4%–99.4%, respectively [10]. However, the changing epidemiology of malaria in low-transmission settings presents new challenges for diagnosis. A higher proportion of asymptomatic infections are subpatent, meaning below the reliable detection limit of RDTs and microscopy, which is a parasite density 100–200/μL [11].

It is well established that for active case detection of asymptomatic infections, RDTs perform poorly [3, 4, 12–15]. However there are limited studies from health facilities (passive case detection) in low-transmission settings. It is presumed that symptomatic *P. falciparum* infections will be patent, because immunity may be lower in low-transmission settings, leading to high levels of parasitemia at presentation. However, it is also possible that with decreased immunity, subjects may become symptomatic at lower parasite densities and thus evade detection by RDT. Additional reasons for compromised diagnostic accuracy of RDTs may include inability to withstand field conditions (ie, high temperatures and humidity), presence of HRP-2 deletions in some populations, and user error, particularly in settings where malaria cases are few and health workers have limited experience and practice [3, 4, 12, 16].

Our study concerns the use of RDTs in patients presenting to health facilities with suspected malaria in the low-transmission setting of Swaziland [17]. RDTs were introduced to all health facilities nationally in 2010, and shortly thereafter, a quality assurance (QA) program was established in the endemic Lubombo region, which involved collection of dried blood spot (DBS) samples in all subjects tested with RDTs for subsequent molecular testing. We investigated the diagnostic accuracy of RDTs using qualitative and quantitative PCR as a reference standard, and used logistic regression models to explore potential factors associated with RDT performance.

## MATERIALS AND METHODS

### Study Design

We conducted a prospective population–based observational study of diagnostic accuracy.

### Study Site

Swaziland is a low-middle–income country in southern Africa. It is a low-transmission setting with a malaria incidence of 0.7–1.3 per 1000 population at risk from 2012 to 2015 and a prevalence of malaria infection last measured at 0.2% in 2010 [17]. The high-transmission season occurs between January and April. Locally acquired cases mostly occur in rural areas in the eastern part of the country, and roughly half of cases are imported, mostly from neighboring Mozambique. *P. falciparum* malaria is the primary species, and the principal vector of disease, *Anopheles arabiensis*, is indoor biting and resting.

### Study Population

The study population included patients with symptomatic malaria cases detected by RDT at all 37 health facilities in the eastern region of Lubombo between August 2012 and April 2014, as well as those with suspected malaria who tested RDT negative. Subjects were excluded only if a DBS sample was inadequate (blood not soaked to back) or missing.

### Data Collection

*P. falciparum*–specific testing was performed using the First Response Malaria Ag *P. falciparium* HRP-2 Detection Rapid Card Test (Premier Medical) according to the manufacturer’s instructions. Different lots of RDTs used in the study passed lot testing at a World Health Organization–qualified international laboratory before arrival in Swaziland. Blood was simultaneously spotted on Whatman 902 filter paper, dried overnight, and stored in sealed plastic bags with desiccant. Training on RDT testing and DBS sample collection was performed annually by the National Malaria Control Programme, with messages reinforced through educational manuals and regular supervisory visits to clinics [18].

RDTs and DBS samples were collected for QA by surveillance officers every 1–3 months and stored at 4°C. DBS samples from all RDT-positive samples and a subset of RDT-negative samples were selected for QA. Sample selection criteria for RDT-negative samples was 10% of negative samples per health facility per month, and ≥1 sample per month from health facilities with any RDT-negative samples. The total number of RDT-negative samples collected for a given facility in a given month was rounded to the nearest tenth, and 10% of this number was randomly selected for analysis. If a facility had 1–4 samples, 1 was selected. The National Malaria Control Programme attempted a follow-up visit with all malaria cases within 48 hours to perform a case investigation, which involved collection of clinical and epidemiological data, including global positioning system coordinates of residence, vector control coverage, and travel history. The QA program included collection of a slide for each RDT-positive case, but these data were omitted owing to frequent improper staining.

### Molecular Testing

DBS samples were transported to the University of California, San Francisco (UCSF), where DNA was extracted using a saponin/Chelex method, as described elsewhere [19]. An established nested PCR (nPCR) method targeting the mitochondrial cytochrome *b* gene was used as the reference standard [20–22]. Species identification for all nPCR-positive samples was conducted using an *Alu*I restriction digest [21].

To explore low-density parasitemia as a potential reason for false-negative RDT results, we measured parasite densities of all nPCR-positive samples using a real-time quantitative PCR (qPCR) method targeting the *Plasmodium* transfer RNA methionine gene [23]. Known density controls were used to create a standard curve. Five microliters of template DNA were used in a 25-µL reaction with the following thermocycling conditions: 95°C for 6 minutes, 60 cycles of: 95°C for 15 seconds, 64°C for 20 seconds, and 68°C for 1 minute.

To explore *pfhrp2* gene deletion as a potential reason for false-negative RDT results, we used extracted DNA from RDT-negative, nPCR-positive samples with parasite densities ≥100/µL to amplify the *pfhrp2* and *pfhrp3* genes per methods published elsewhere [24, 25].

### Data Management and Analysis

RDT data from health facilities were collected on paper then entered into Microsoft Excel, merged with molecular data, and cleaned and analyzed using Stata software (version 14.0). RDT and PCR results were compared, and the diagnostic accuracy of RDTs was calculated first, using nPCR as the reference standard. Because the known detection limit of RDTs was a parasite density of about 100/µL, diagnostic accuracy was also calculated excluding low-density samples (<100/µL). For sensitivity and specificity, the Begg and Greens method was used to calculate confidence intervals (CIs), to account for verification bias that may have occurred as a result of sampling a proportion of RDT-negative samples [26, 27].

Other potential reasons for RDT false-negativity or false-positivity (RDT testing volume and number of RDT-positive samples by health facility, transmission season, and transmission year) were explored by means of χ^2^ analyses for categorical data or *t* tests for continuous variables and logistic regression models. Covariates were included in the multivariate analysis if the relationship in the bivariate analysis was significant (95% CI not including 1.0). Potential DNA degradation from delayed PCR processing (measured as days from sample collection to PCR testing) as well as demographic, clinical, behavioral, and epidemiological data obtained from case investigations were similarly tested for associations with false-positivity.

### Ethics

Ethical approval was obtained from UCSF and the Swaziland Ministry of Health.

## RESULTS

### Recruitment

**Figure 1.**
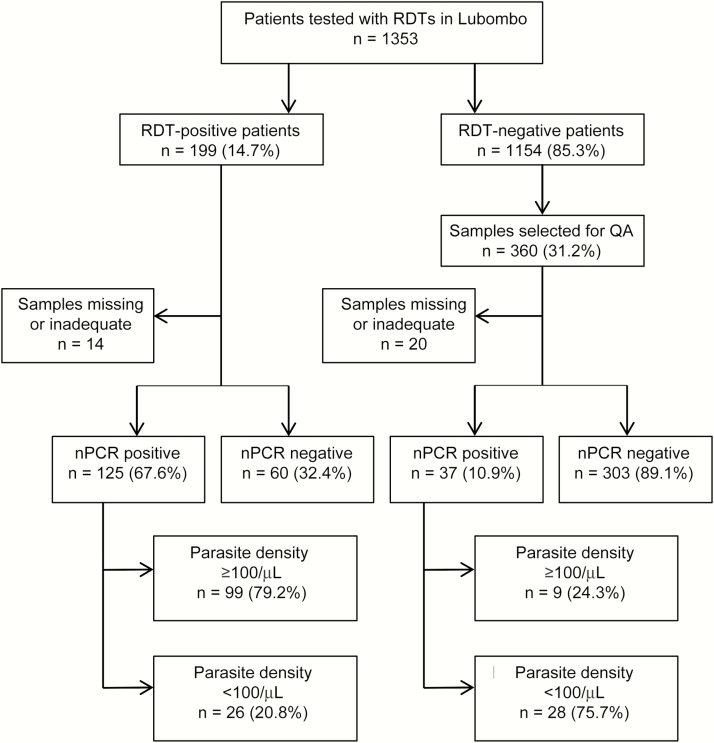
Flow chart of study participant recruitment with rapid diagnostic tests (RDTs), nested PCR (nPCR), and quantitiative PCR results. Abbreviation: PCR, polymerase chain reaction; QA, quality assurance.

A total of 1353 patients with suspected malaria were tested with RDTs between June 2012 and April 2014 in 37 health facilities in the Lubombo region (Figure 1). Of 199 RDT-positive and 1154 RDT-negative samples, 31.2% were selected for QA. Thirty-four DBS samples were either missing or contained an inadequate amount of blood.

### Qualitative PCR and qPCR Results

Among RDT-positive patients, 67.7% were nPCR positive; among RDT-negative patients, 10.9% were nPCR positive. Of the 162 nPCR-positive samples, 160 were classified as *P. falciparum*. Two RDT- and nPCR-positive samples could not be speciated.

**Figure 2.**
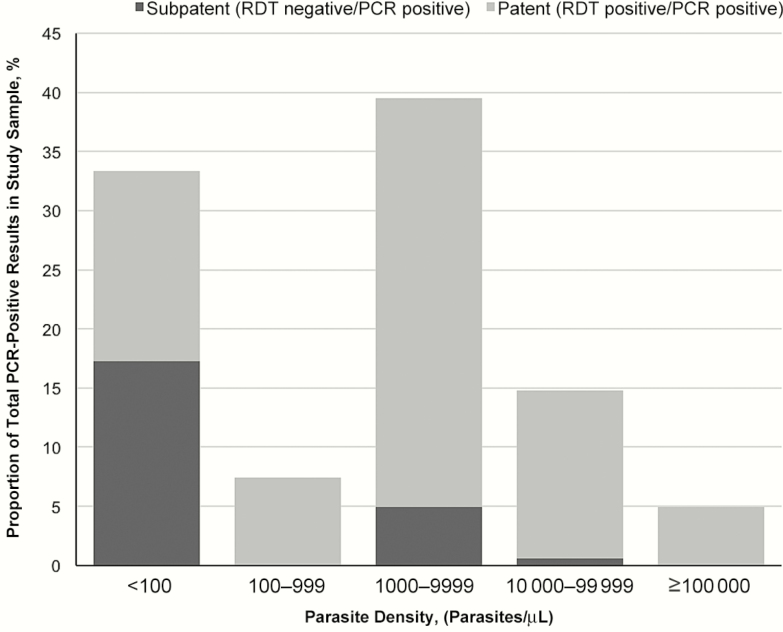
Parasite densities of nested PCR (PCR)–positive samples and proportion of subpatent and patent by parasite density category. Abbreviation: PCR, polymerase chain reaction; RDT, rapid diagnostic test.

Distributions of parasite densities as determined by qPCR are shown in Figure 2. In 33.3% of PCR-positive samples, the parasite density was <100/µL and thus below the standard limit of detection for RDTs. The parasite density was 100–99999/µL in 61.7% and ≥100000/µL in 4.9%. Subpatent infections represented 17%, 9%, and 0% of samples with parasite densities of <100, 100–99999, and ≥100000/µL, respectively.

### Diagnostic Accuracy of RDT With nPCR as Reference Standard

Table 1 displays all RDT and nPCR results (n = 525) as well as RDT and nPCR results excluding samples with parasite density <100/µL (n = 471). The false-positive and false-negative rates were 32.4% (60 of 185) and 10.9% (37 of 340), respectively. The sensitivity of RDT with nPCR used as the reference standard was 51.7% (95% CI, 42.9%–60.4%), compared to 78.8% (65.5%–87.9%) after exclusion of samples with a parasite density <100/µL (Table 2). Negative predictive value (NPV) improved from 89.1% to 97.1% when low-density samples were excluded. In both analyses, specificity was high, and the positive predictive value (PPV) was low.

**Table 1.** A comparison of RDT and nPCR Results Among All Samples and Excluding Low-Density Infections

Sample Group	nPCR Positive, No.	nPCR Negative, No.	Total, No.
All samples			
RDT positive	125	60	185
RDT negative^a^	37	303	340
Total	162	363	525
Excluding samples with parasite density <100/µL			
RDT positive	99	60	159
RDT negative^a^	9	303	312
Total	108	363	471

Abbreviations: nPCR, nested polymerase chain reaction; RDT, rapid diagnostic test.

^a^RDT-negative samples with nPCR results represented 31.2% of all RDT-negative samples selected for quality assurance.

**Table 2.** Diagnostic Accuracy of RDTs using nPCR as a Reference Standard

RDTs	Value (95% CI), %			
	Sensitivity	Specificity	PPV	NPV
All samples	51.7 (42.9–60.4)	94.1 (92.5–95.4)	67.3 (60.3–73.8)	89.1 (87.1–90.8)
Excluding samples with parasite density <100/µL	78.8 (65.5–87.9)	93.7 (92.1–95.0)	62.3 (55.2–69.1)	97.1 (96.0–98.0)

Abbreviations: CI, confidence interval; nPCR, nested polymerase chain reaction; NPV, negative predictive value; PPV, positive predictive value; RDT, rapid diagnostic test.

### Absence of *pfhrp2* and *pfhrp3* as Potential Cause of False-Negative RDT Results

Among 9 RDT false-negative samples with parasite density >100/µL (median, 3954; range 1277–13889/µL), *pfhrp2* gene amplified in all samples, and *pfhrp3* amplified in all but 1 sample that also did not amplify in a flanking region of *pfhrp2* (mal7p1230).

### Other Factors Associated With RDT False-Negativity or False-Positivity

A total of 9 false-negative samples were compared with 303 true-negative samples in terms of RDT testing volume and number of RDT-positive samples by health facility, transmission season, and transmission year, and no significant associations were found (data not shown).

RDT true-positive samples (n = 125) were compared with false-positive samples (n = 60) in terms of RDT testing volume and number of RDT-positive samples by health facility, transmission season, and year (Table 3). In the multivariate analysis, only 2013–2014 transmission year (vs the prior year), was associated with false-positivity (adjusted odds ratio, 2.41; 95% CI, 1.17–4.97). To explore DNA degradation from delayed PCR processing as a potential cause of an incorrect false-positive classification, RDT-positive/PCR-negative samples were compared to RDT-positive/PCR-positive samples, and the mean (standard deviation) processing time was 235 (89) versus 270 (116) days, respectively (*P* = .04). Finally, detailed data from case investigations was linked to QA data for 77 of the 125 RDT true-positive (61%) and 32 of the 60 RDT false-positive (53%) samples. We explored age, sex, nationality, occupation, time from symptom onset to seeking treatment, severity of disease, reported history of malaria in the prior year, travel outside the country or within Swaziland in the past 8 weeks, bed net usage, indoor residual spraying coverage, housing quality, distance to a water body, and elevation, and none of these variables was significantly associated with false-positivity (data not shown).

**Table 3.** Measuring Associations Between Potential Epidemiological Factors and RDT False-Positivity

Variable	Results, No. (%)		OR (95% CI)	aOR (95% CI)
	False- Positive (n *=* 60)	True- Positive (n = 125)		
Health facility testing volume during study period, No. of RDTs				
≤2	1 (25.0)	3 (75.0)	1.00 (Reference)	NA
>2 and ≤ 9	5 (35.7)	9 (64.3)	1.67 (.13–20.58)	
>9 and ≤30	22 (48.9)	23 (51.1)	2.87 (.28–29.71)	
>30	32 (26.2)	90 (73.8)	1.07 (.11–10.63)	
Health facility positive volume, No. of RDT-positive samples during study period				
1	4 (57.1)	3 (42.9)	1.00 (Reference)	NA
2	2 (20.0)	8 (80.0)	0.19 (.02–1.62)	
3–6	9 (52.9)	8 (47.1)	0.84 (.14–4.97)	
9–64	45 (35.7)	81 (64.3)	0.42 (.09–1.94)	
Transmission season				
January–April	31 (27.0)	84 (73.0)	1.00 (Reference)	1.64 (.86–3.14)
May–December	29 (41.4)	41 (58.6)	1.92 (1.02–3.60)	
Transmission year				
2012–2013	13 (19.7)	53 (81.3)	1.00 (Reference)	2.41 (1.17–4.97)
2013–2014	40 (39.5)	72 (60.5)	2.66 (1.30–5.41)	

Abbreviations: aOR, adjusted odds ratio; CI, confidence interval; NA, not applicable; OR, odds ratio; RDT, rapid diagnostic test.

## DISCUSSION

Using qualitative PCR and qPCR as a reference standard, we evaluated the diagnostic accuracy of HRP-2 RDTs used for suspected malaria at 37 health facilities over a 2-year period in the low-transmission setting of Swaziland. Specificity and NPV were acceptable, but sensitivity and PPV were low, at 51.7% and 67.3%, respectively. Sensitivity improved to 78.8% when the low-density infections were excluded. False-positivity was more likely during the second transmission season and was not associated with other factors such as health facility testing characteristics, DNA degradation (as measured by time to PCR processing), demographics, or recent malaria, which has been implicated as the main reason for false-positive results in higher-transmission settings.

Prior studies have shown high sensitivity and specificity of RDTs. In a Cochrane meta-analysis that included 84 evaluations of HRP-2 RDTs, the average sensitivity and specificity were 95.0% and 95.2%, respectively [10]. The false-positive rate of HRP-2 RDTs was acceptable at 5% but slightly higher than that of other antigen-based RDTs, attributed to the fact that HRP-2 antigen can persist for 28 days even after effective treatment. However, the review was limited, in that there were no exclusively low-transmission (prevalence, <10%) study sites that used PCR as the reference standard.

In our study, low parasite density was the predominant reason for false-negative RDT results (75.7%). For the remaining 24.3%, deletion of *pfhrp2* or *pfhrp3* genes was ruled out as a cause of false-negativity [24, 28]. A prozone effect of hyperparasitemia/antigen overload leading to false-negative results [29] was not likely because the parasite densities among these 9 samples were not high (mean, 4546/µL; range, 1277–13889/µL). Conversely, low circulating levels of the HRP-2 antigen among patients presenting with symptoms early in infection may have contributed to the results. Finally, an indirect assessment of RDT product quality, transport or storage conditions, and user error (considering at health facility RDT testing volume, season, and year) was unrevealing.

Also concerning was the high false-positive rate (32.4%), which compromised PPV owing to the low prevalence of infection in this setting. We considered DNA degradation, because loss of sensitivity has been reported for samples stored ≥2 years at ambient temperature [22]. However, for RDT-positive samples, processing time was not longer for PCR-negative versus PCR-positive samples, and all samples except 1 were processed within 2 years. Antigenemia among subjects with recent infection cleared through immunity or treatment is the most common reason for false-positive RDT results in moderate- and high-transmission settings, but this explanation seems unlikely in our low-transmission settings. Furthermore, compared with true-positivity, false-positivity was not associated with diagnosis of malaria in the past year or other potential indicators of recent infection, such as age, sex, occupation, Mozambican nationality, length of symptoms, recent travel, and vector control coverage. False-positivity was more likely during the second half of the study, but for unclear reasons. The 1.5 times higher case load during the second year may have led health workers to over-read RDT results as positive, or there may been other time-specific user error, storage, or transport issues [30]. Improved training and supervision, as well as quality control (eg, through the use of positive control wells), may be needed [8]. RDT product quality seems a less likely cause, given lot testing of RDTs before arrival in Swaziland.

Our study had some limitations. Unlike in most prior studies evaluating the diagnostic accuracy of RDTs, we were unable to use microscopy as a reference standard. However, owing to limitations of microscopy, molecular methods are now recommended for QA of RDTs [9], and our qPCR-corrected analysis enables comparison with past studies while also validating the nPCR results. Measurement of HRP-2 antigen levels, versus DNA, as a more comparable analyte to the RDT, may have been useful, but these methods have not been standardized and would not have been easily performed within the context of a national QA program. Owing to selection of negative samples, we may have over- or underselected for false-negative samples, but we did adjust for verification bias [27]. Future efforts could include all negative samples, but it may not be feasible to process a higher volume of samples. We were limited in our ability to assess reasons for false-positivity. Future assessments could be improved by collecting detailed quality control data (eg, storage and transport conditions), qualitative study of reasons for overdiagnosis [31], and integrating the QA and case investigation programs to enable more complete linking of individual case data. Finally, owing to the low malaria incidence in Swaziland, there were few RDT-positive samples, and CIs for the estimates of sensitivity were wide. Additional studies from other low-transmission settings should be pursued.

Our study had several strengths. We had a large regional data set that captured 1353 subjects from 37 health facilities and covered 2 years. To our knowledge, our is the first study from an exclusively low-endemicity setting (parasite prevalence by PCR, <10%) to use PCR to assess the diagnostic performance of HRP-2 RDTs among patients with suspected malaria. We employed additional robust nucleic acid methods (qPCR and PCR targeting *pfhrp2/3* genes) to implicate low-density infection as the primary reason for low sensitivity. Moreover, our findings of low sensitivity and PPV have important implications for both clinical practice and public health. Missed or delayed diagnoses can lead to disease progression, underestimates of disease burden, and missed opportunities for active surveillance in the communities of index cases [15]. Likewise, false-positives can lead to overtreatment and inflated estimates of disease burden.

Our study also has important implications for low-transmission/malaria elimination settings. First, a higher than expected proportion of suspected malaria cases were low-density (33.3%), suggesting that clinical malaria may present at lower parasite densities in lower-transmission settings [32]. The parasite density pyrogenic threshold for *P. falciparum* malaria has been found to be as low as 10/µL [33]. Alternatively, these infections may not represent the primary disease, and it has been argued that identification of incidental low-density parasitemia may distract from appropriate management of severe nonmalarial illnesses. However, when the goal is malaria elimination, all infections have potential to seed transmission and warrant treatment, and health workers should be trained to manage a more nuanced and broader differential diagnosis of febrile illness [34]. Owing to processing time and resource constraints, PCR using DBS samples is impractical for clinical purposes in endemic settings [35]. More sensitive point-of-care diagnostics are in development. The World Health Organization has not called for their use in clinical settings, as it has for the detection of asymptomatic infections in active surveillance [9], but our findings suggest that they may be necessary in low-endemicity settings. We conclude that a more sensitive and specific point-of-care diagnostic, as well as improved quality control and assurance to address over- or underdiagnosis, is needed to support malaria clinical case management and broader elimination efforts in Swaziland, and potentially in other low-transmission settings.

## Notes

***Acknowledgments.*** We thank Joe Novotny at the Clinton Health Access Initiative for support in field coordination; Cebsile Shabalala, Sindi Dlamini, and Maphalala Gugu at the National Reference Laboratory for laboratory support; Joe Vinetz at the University of California, San Diego, and Dionica Gamboa at Universidad Peruana Cayetano Heredia for sharing the *pfhrp2/3* protocols; Danica Helb at UCSF for laboratory advice; Grant Dorsey, Kimberly Baltzell, Madhavi Dandu, Phil Rosenthal, and Roly Gosling at UCSF for their guidance; Theodoor Visser at the Clinton Health Access Initiative for his review of the manuscript; health facility personnel at all sites; and study participants.

***Financial support.*** This work was supported by the Swaziland Ministry of Health; the Bill and Melinda Gates Foundation (grant A121292); the National Institute of Allergy and Infectious Diseases, National Institutes of Health (grant AI101012 to M. S. H.); Burroughs Wellcome Fund/American Society for Tropical Medicine and Hygiene Fellowship (grant A120079 to M. S. H.); the Horchow Family Fund (grant 5300375400 to M. S. H.); and the Gilead Translational Fellows (grant NCE A122737 to N. R.).

***Potential conflicts of interest.*** All authors: No reported conflicts. All authors have submitted the ICMJE Form for Disclosure of Potential Conflicts of Interest. Conflicts that the editors consider relevant to the content of the manuscript have been disclosed.
